# Qualitative Analysis of Tumor-Infiltrating Lymphocytes across Human Tumor Types Reveals a Higher Proportion of Bystander CD8^+^ T Cells in Non-Melanoma Cancers Compared to Melanoma

**DOI:** 10.3390/cancers12113344

**Published:** 2020-11-12

**Authors:** Aishwarya Gokuldass, Arianna Draghi, Krisztian Papp, Troels Holz Borch, Morten Nielsen, Marie Christine Wulff Westergaard, Rikke Andersen, Aimilia Schina, Kalijn Fredrike Bol, Christopher Aled Chamberlain, Mario Presti, Özcan Met, Katja Harbst, Martin Lauss, Samuele Soraggi, Istvan Csabai, Zoltán Szállási, Göran Jönsson, Inge Marie Svane, Marco Donia

**Affiliations:** 1 National Center for Cancer Immune Therapy (CCIT-DK), Department of Oncology, Copenhagen University Hospital, 2730 Herlev, Denmark; aishwarya.gokuldass@regionh.dk (A.G.); arianna.draghi.01@regionh.dk (A.D.); troels.holz.borch@regionh.dk (T.H.B.); morten.nielsen.03@regionh.dk (M.N.); marie.christine.wulff.westergaard@regionh.dk (M.C.W.W.); Rikke.Andersen.02@regionh.dk (R.A.); aimilia.schina@regionh.dk (A.S.); kalijn.fredrike.bol@regionh.dk (K.F.B.); christopher.aled.chamberlain@regionh.dk (C.A.C.); mario.presti@regionh.dk (M.P.); Ozcan.Met@regionh.dk (Ö.M.); Inge.Marie.Svane@regionh.dk (I.M.S.); 2 Department of Physics of Complex Systems, ELTE Eötvös Loránd University, H-1117 Budapest, Hungary; pkrisz5@elte.hu (K.P.); csabai@complex.elte.hu (I.C.); 3 Department of Immunology and Microbiology, Faculty of Health and Medical Sciences, University of Copenhagen, 2200 Copenhagen, Denmark; 4 Department of Clinical Sciences Lund, Division of Oncology and Pathology, Faculty of Medicine, Lund University, 221 00 Lund, Sweden; katja.harbst@med.lu.se (K.H.); martin.lauss@med.lu.se (M.L.); goran_b.jonsson@med.lu.se (G.J.); 5 Lund University Cancer Centre, Lund University, 221 00 Lund, Sweden; 6 Bioinformatics Research Center, Aarhus University, 8000 Aarhus, Denmark; samuele@birc.au.dk; 7 Danish Cancer Society Research Center, 2100 Copenhagen, Denmark; Zoltan.Szallasi@childrens.harvard.edu

**Keywords:** tumor-infiltrating lymphocytes, tumor microenvironment, immunotherapy

## Abstract

**Simple Summary:**

Human tumors are often infiltrated by T cells; however, it remains unclear what proportion of T cells infiltrating tumors are bystander and non-tumor specific. We have investigated qualitative characteristics of these tumor-infiltrating lymphocytes (TILs) based on their gene-expression in the tumor-microenvironment or on their response to autologous tumor cells in vitro. Despite a considerable inter-sample variability, we found the overall proportion of bystander (non-tumor reactive) TILs to be remarkably high. Importantly, we observed a higher proportion of bystander TILs in non-melanoma tumors, compared to melanoma. This study suggests that immunotherapeutic strategies, especially when applied to non-melanoma tumors, should be tailored to reinvigorate the small proportion of tumor-reactive T cells infiltrating the tumor-microenvironment.

**Abstract:**

*Background:* Human intratumoral T cell infiltrates can be defined by quantitative or qualitative features, such as their ability to recognize autologous tumor antigens. In this study, we reproduced the tumor-T cell interactions of individual patients to determine and compared the qualitative characteristics of intratumoral T cell infiltrates across multiple tumor types. *Methods:* We employed 187 pairs of unselected tumor-infiltrating lymphocytes (TILs) and autologous tumor cells from patients with melanoma, renal-, ovarian-cancer or sarcoma, and single-cell RNA sequencing data from a pooled cohort of 93 patients with melanoma or epithelial cancers. Measures of TIL quality including the proportion of tumor-reactive CD8^+^ and CD4^+^ TILs, and TIL response polyfunctionality were determined. *Results:* Tumor-specific CD8^+^ and CD4^+^ TIL responses were detected in over half of the patients in vitro, and greater CD8^+^ TIL responses were observed in melanoma, regardless of previous anti-PD-1 treatment, compared to renal cancer, ovarian cancer and sarcoma. The proportion of tumor-reactive CD4^+^ TILs was on average lower and the differences less pronounced across tumor types. Overall, the proportion of tumor-reactive TILs in vitro was remarkably low, implying a high fraction of TILs to be bystanders, and highly variable within the same tumor type. In situ analyses, based on eight single-cell RNA-sequencing datasets encompassing melanoma and five epithelial cancers types, corroborated the results obtained in vitro. Strikingly, no strong correlation between the proportion of CD8^+^ and CD4^+^ tumor-reactive TILs was detected, suggesting the accumulation of these responses in the tumor microenvironment to follow non-overlapping biological pathways. Additionally, no strong correlation between TIL responses and tumor mutational burden (TMB) in melanoma was observed, indicating that TMB was not a major driving force of response. No substantial differences in polyfunctionality across tumor types were observed. *Conclusions:* These analyses shed light on the functional features defining the quality of TIL infiltrates in cancer. A significant proportion of TILs across tumor types, especially non-melanoma, are bystander T cells. These results highlight the need to develop strategies focused on the tumor-reactive TIL subpopulation.

## 1. Introduction

The success of cancer immunotherapy relies on the activation of a potent effector T cell response. These antitumor immune responses mediate tumor regression via recognition of tumor antigens presented on the surface of tumor cells by major histocompatibility complex (MHC) molecules. The most successful immune responses are believed to target antigens deriving from somatic tumor mutations, or neo-antigens, recognized with exquisite specificity by the T cell receptors (TCRs) expressed by tumor-infiltrating lymphocytes (TILs) [1]. An abundance of somatic tumor mutations (high tumor mutational burden, or TMB) translates into a high number of neo-antigens [2]. Immunologically active or “hot” tumors [3], especially those bearing a high TMB, are particularly sensitive to treatments stimulating adaptive immunity, such as cancer immunotherapy with immune checkpoint inhibitors [4]. Recent studies have demonstrated that TMB does not correlate well with the intratumoral immune activity of a given cancer when measured with standard methods, suggesting these two parameters to be independent.

Hot tumors are commonly defined by unspecific immunologic measures quantifying intratumoral immune activity, such as broad immune response gene-signatures related to T cell infiltration or interferon gamma (IFNγ) activity [5,6,7,8] and PD-L1 expression [9]. However, there is currently no singular definition of hot tumors. Recent in-depth TCR characterization of TILs in distinct tumor types revealed that the majority of CD8^+^ TILs do not have the potential to recognize and kill autologous tumor cells ([10,11]). A fraction of CD8^+^ TILs can be bystanders and recognize non-tumor related viral antigens [11,12], whereas CD4^+^ TILs can be both bystander [13] and/or forkhead Box P3 (FOXP3)^+^ tumor-specific T cells [14], which may be endowed with regulatory T cell functions. Activating non-tumor specific or regulatory T cells with immunotherapy, even in cases of significant T cell infiltration, is unlikely to induce tumor regression. These data have highlighted the issue of correctly identifying bona fide hot tumors, which are characterized not only by high immune cell infiltration but also an active tumor-directed immune response. Hence, novel approaches to study both the quantity and the quality of the tumor immune infiltrate at the functional level are highly warranted. The quality of a tumor immune infiltrate can be measured by the ability of TILs to recognize autologous tumor antigens and carry out functions such as type 1 cytokine secretion [15], mobilization of cytotoxic granules [16], or upregulation of costimulatory molecules [17].

In this study, we addressed this issue by measuring qualitative features of the TIL infiltrates based on recognition of autologous tumor antigens. This led to the identification of the proportion of tumor-reactive TILs and their corresponding functional profiles. We studied the association of TIL quality parameters to immunological and genomic biomarkers across multiple tumor types representative of the broad TMB spectrum. These data can serve as a reference for any future study describing the level of functional antitumor reactivity of a given population of tumor-reactive T cells.

## 2. Results

### 2.1. Antitumor Reactivity In Vitro: Testing Modalities 

A total of 187 TILs/tumor cell pairs were obtained over a decade from individual patients spanning four distinct tumor types and five clinical cohorts (Table S1). Due to the large time frame of sample acquisition and analysis, as well as technical differences between cohorts, preliminary analyses were carried out to check for potential confounding variables and to estimate the comparability of all cohorts. The success rates of tumor cell line (TCL) generation that we reported previously were distinct across tumor types (Metastatic Melanoma (MM) 61% (consisting of two sub groups named as MM PD-1 naïve, including samples deriving from patients who were not treated with anti-PD1 previously, and MM PD-1 resistant (MM PD-1 res), including samples deriving from patients who were treated with anti-PD1 and progressed), renal cell carcinoma (RCC) 77%, ovarian cancer (OC) 32%, and sarcoma (SAR) 50% [18,19,20,21,22]), hence, different proportions of samples across cohorts were tested with fresh tumor digests (FTDs) only, or with autologous TCLs pre-treated or not with recombinant human IFNγ (Tables S2 and S3). A pooled analysis from all pairs that were tested with both TCLs/TCLs + IFNγ and FTDs (*n* = 71; 22 MM, 19 RCC, 21 OC and 9 SAR) showed that tests against FTDs yielded a lower reactivity for CD8^+^ (*p* = 0.018, Figure S1A) and a higher reactivity for CD4^+^ TILs (*p* = 0.039, Figure S1B) compared to testing against TCLs/TCLs + IFNγ. These data could indicate additional tumor-antigen presentation by non-tumor cells (e.g., stromal- or antigen presenting-cells) to CD4^+^ T cells via MHC class II in assays using FTDs. When testing for potential differences in reactivity among pairs tested separately with Young TILs (Y TILs) and Rapidly Expanded TILs (REP TILs) (Tables S2 and S3), no significant differences were observed in CD8^+^ (*n* = 132; 59 MM, 28 RCC and 45 OC; *p* = 0.25, Figure S1C) or CD4^+^ (*n* = 128; 55 MM, 28 RCC and 45 OC; *p* = 0.66, Figure S1D) T cell reactivity. These data indicate that massive TIL-expansion with the rapid expansion protocol (REP) does not significantly impair the proportion of tumor-reactive TILs. 

Overall, the differences in testing with TCLs/TCLs + IFNγ versus FTDs were statistically significant yet minor when compared to the larger differences observed when comparing cohorts. In addition, it cannot be ruled out that the ability to establish a TCLs is associated with a specific pattern of reactivity. Hence, we report pooled analyses using TCLs/TCLs + IFNγ or FTDs (showing only the highest reactivity), as well as data where only TCLs/TCLs + IFNγ were used.

### 2.2. Antitumor Reactivity In Vitro across Clinical Cohorts

**Figure 1:**
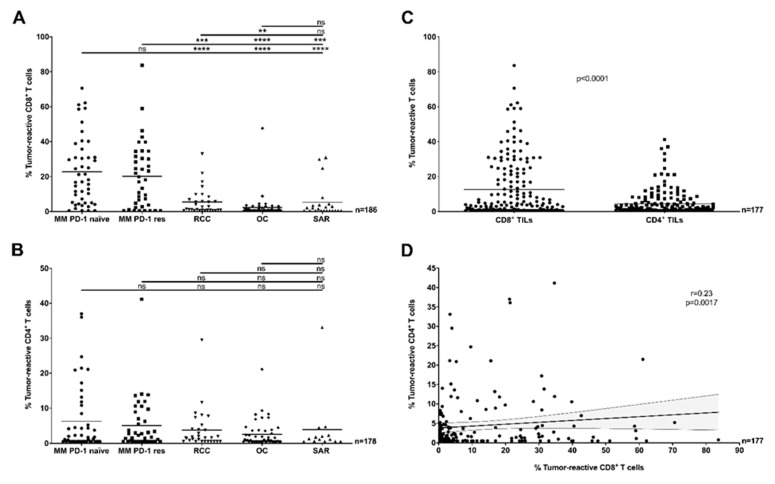
Antitumor-reactivity of tumor-infiltrating lymphocytes (TILs) across clinical cohorts and TIL sub-populations (in vitro, pooled data). (**A**) The proportion of tumor-reactive CD8^+^ TILs was significantly greater in Metastatic Melanoma (MM) cohorts compared to other tumor types, with no difference related to previous exposure to anti-PD-1 therapy (Mann–Whitney U test, *p* < 0.001). (**B**) The proportion of tumor-reactive CD4^+^ TILs was similar across all clinical cohorts (Mann–Whitney test, *p* > 0.05). (**C**) Comparing TIL subpopulations demonstrated that a greater proportion of CD8^+^ TILs were tumor-reactive compared to CD4^+^ TILs (Mann–Whitney test *p* < 0.0001, pooled clinical cohorts). (**D**) The proportion of tumor-reactive CD8^+^ TILs was only weakly correlated (Spearman *r* = 0.23, *p* = 0.0017) to the proportion of tumor-reactive CD4^+^ TILs in the same samples (pooled clinical cohorts). The solid line and dotted lines represent the best-fit regression line and 95% confidence interval, respectively. (**A**–**D**) In all panels, the recognition of TILs (Young TILs (Y TILs) and Rapidly Expanded TILs (REP TILs)) was tested against separate sets of autologous tumor cells (tumor cell lines (TCLs), TCLs + interferon gamma (IFNγ) or fresh tumor digests (FTDs)) and only the highest value reported. T cells were considered reactive if positive for at least one of TNF, IFNγ or CD107a, minus control. ** *p* < 0.01, *** *p* < 0.001, **** *p* < 0.0001, ns: no statistical significance.

In pooled analyses with TCLs/TCLs + IFNγ and FTDs (*n* = 186, Figure 1A), the mean proportion of tumor-reactive CD8^+^ TILs of melanoma samples, regardless of previous anti-PD-1 therapy, far surpassed other cohorts (*p* < 0.001). Except RCC being greater than OC (*p* < 0.01), the other cohorts were highly similar to each other. However, there was considerable inter-sample variability within each clinical cohort, and individual samples in each of the other cohorts exceeded the mean reactivity level of CD8^+^ TILs in MM. Of note, CD8^+^ tumor-reactive TILs (above the detection limit of 0.5%) were detected in around 50% or more samples within each clinical cohort (range 50% in OC to 91% in MM PD-1 naïve). Analysis of reactivity against only TCLs/TCLs + IFNγ confirmed this pattern, except differences in non-melanoma (non-MM) tumors were no longer present (*n* = 143, Figure S2A).

The downstream effects of direct recognition of tumor-antigens by tumor-reactive CD4^+^ TILs have not been well documented so far. Here, in pooled analyses with TCLs/TCLs + IFNγ and FTDs (*n* = 177), the proportion of tumor-reactive CD4^+^ TILs was similar across all cohorts, and tumor-specific CD4^+^ T cell responses were detected in over 50% of patients in each clinical cohort (range 58% in OC to 71% in RCC) (Figure 1B). A high inter-sample variability within individual cohorts was observed. When only responses to TCLs/TCLs + IFNγ were considered, responses were increased in MM PD-1 res samples compared to RCC (*p* < 0.05), in both melanoma cohorts (regardless of previous anti-PD-1 treatment) compared to OC (*p* < 0.001) and in SAR compared to OC (*p* < 0.01) (*n* = 135, Figure S2B). Differences between MM and non-MM tumors were considerably less pronounced compared to CD8^+^ TIL responses. 

In order to assess whether the differences in the proportion of tumor-reactive CD8^+^ TILs observed in distinct tumor types were associated with other parameters of importance for T cell-mediated recognition of tumor-antigens, we re-analyzed data from The Cancer Genome Atlas (TCGA) and compared TCR richness [23] and antigen processing and presentation (APM) machinery activity across tumor types. Both parameters were not significantly increased in MM samples when compared to other tumor types (Figure S3A,B), hence neither of these parameters could explain the higher antitumor-reactivity of CD8^+^ TILs observed in MM.

Interestingly, when comparing the proportion of tumor-reactive CD8^+^ TILs to CD4^+^ TILs, a significant difference in favor of CD8^+^ TIL responses was observed when pooling all cohorts together (*p* < 0.0001, *n* = 177, Figure 1C). Segregation by cohort revealed that these differences were largely driven by MM (Figure S4), regardless of previous anti-PD-1 therapy. In addition, we found only a weak correlation linking the proportion of tumor-reactive CD8^+^ and CD4^+^ TILs in each sample (*r* = 0.23, *p* < 0.0017, *n* = 177, Figure 1D). 

### 2.3. Antitumor Reactivity In Situ

**Figure 2:**
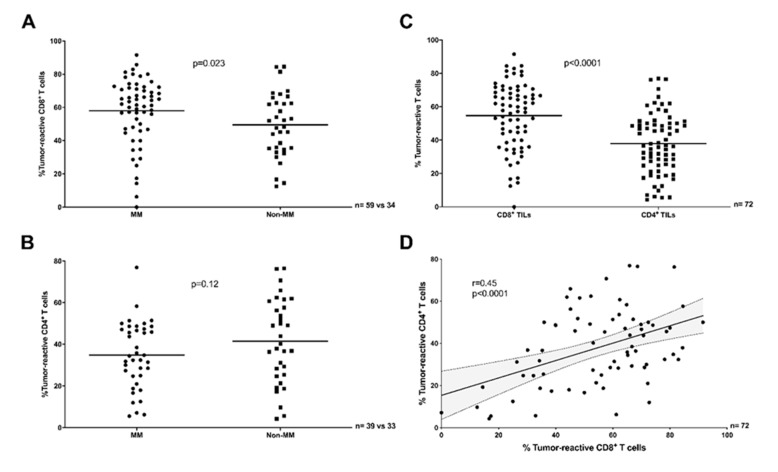
Antitumor-reactivity of TILs across clinical datasets and TIL sub-populations (scRNAseq in situ). (**A**) The proportion of tumor-reactive CD8*+* TILs was greater in MM (PD-1 naïve plus PD-1 res) compared to non-MM (Mann–Whitney test, *p* = 0.023). (**B**) The proportion of tumor-reactive CD4*+* TILs was comparable in MM (PD-1 naïve only) and non-MM (Unpaired *t* test, *p* = 0.12). (**C**) A greater proportion of CD8*+* TILs were tumor-reactive compared to CD4*+* TILs (Paired *t* test, *p* < 0.0001, MM PD-1 naïve plus non-MM). (**D**) The proportion of tumor-reactive CD8*+* TILs was only moderately correlated to the proportion of tumor-reactive CD4*+* TILs in the same samples (Pearson *r* = 0.45, *p* < 0.0001, MM PD-1 naïve plus non-MM). The solid line and dotted lines represent the best-fit regression line and 95% confidence interval, respectively. (A–D) T cells were considered reactive if positive for the expression of least one of *TNF*, *IFNG*, or *TNFRSF9*.

In vitro studies with expanded TILs may not reflect the exact proportion of tumor-reactive T cells found in situ in the TME, as TIL expansion can result in culture-induced changes in clonal composition [24]. In order to further investigate the varying proportions of truly tumor-reactive TILs between distinct tumor types in situ, we re-analyzed single cell RNA-sequencing (scRNAseq) data from 101 tumor biopsies of 93 patients with MM [25,26,27] or non-MM epithelial [28,29,30,31,32] tumor types. After merging multiple datasets, we identified a total of 16,651 CD8^+^ and 14,036 CD4^+^ TILs and based on the gene expression of functional markers related to recent T cell activation (see Section 4.6) determined the proportion of tumor-reactive CD8^+^ TILs (Figure 2A) or CD4^+^ TILs (Figure 2B and Figure S5B) in MM versus non-MM epithelial samples, the proportion of tumor-reactive CD8^+^ versus CD4^+^ TILs (Figure 2C), and the correlation in the proportion of tumor-reactive CD8^+^ and CD4^+^ TILs in each sample (Figure 2D). These analyses largely reproduced the results obtained in vitro, shown in Figure 1, differing only in the numerically greater number of tumor-reactive TILs detected in situ, and in a marginally stronger (*r* = 0.47, Figure 2D) positive correlation of CD8^+^ and CD4^+^ TIL-responses.

### 2.4. Polyfunctionality of Responses In Vitro across Tumor Types

We recently demonstrated that tumor-reactive CD8^+^ T cells derived from melanoma and renal cancer can be characterized by their functional patterns [20]. Therefore, we expanded this analysis by determining whether tumor-reactive TILs derived from multiple tumor types were endowed with distinct (poly)functional profiles. 

The functional profile of CD8^+^ and CD4^+^ TILs isolated from the two cohorts of MM, anti-PD-1 naïve and MM PD-1 res, did not significantly differ (*p* = 0.9 and *p* = 0.3, Figure S6). Hence, additional analyses were performed comparing the four distinct tumor types regardless of previous anti-PD-1 therapy. Consistent with our previous data from a smaller but partially overlapping cohort [20], tumor-reactive CD8^+^ T cells isolated from MM exhibited greater polyfunctionality compared to CD8^+^ T cells isolated from RCC and, additionally, from SAR (Figure S7). However, these differences were not significant in all other tumor types comparisons (Figure S7). No major differences were observed within the CD4^+^ tumor-reactive TILs, which appeared to be primarily characterized by tumor necrosis factor (TNF) production only, and a smaller population releasing both IFNγ and TNF (Figure S8). Overall, our analysis did not depict extensive differences in T cell-polyfunctional profiles across the tumor panel analyzed.

### 2.5. Tumor-Reactive TILs In Vitro and TMB in Melanoma

**Figure 3:**
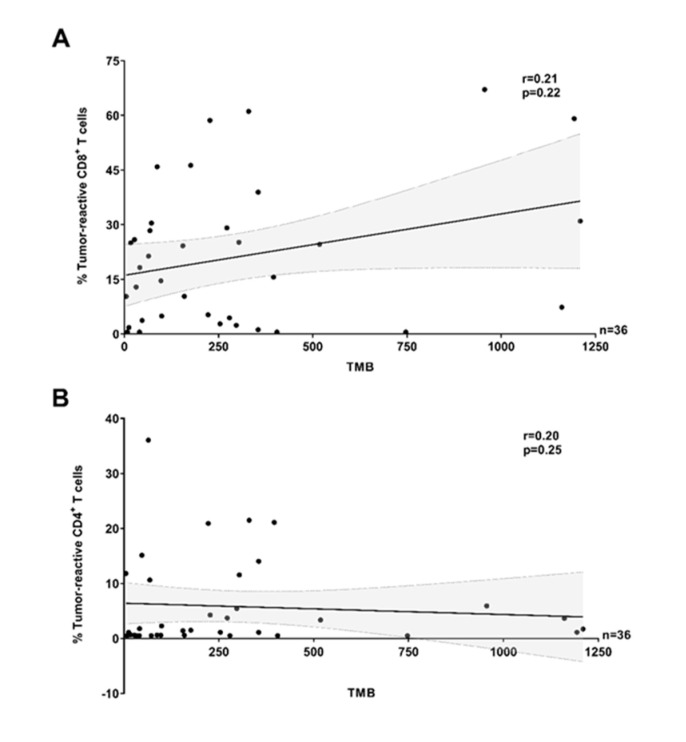
Antitumor-reactivity of TILs and tumor mutational burden in melanoma (pooled data). The proportion of tumor-reactive (**A**) CD8^+^ and (**B**) CD4^+^ TILs was not correlated (Spearman *r* = 0.21, *p* = 0.22 and Spearman *r* = 0.20, *p* = 0.25, respectively) to tumor mutational burden (TMB) (pooled MM clinical cohorts). The solid lines and dotted lines represent the best-fit regression line and 95% confidence interval, respectively. (**A**,**B**) In all panels, the recognition of TILs (Y TILs and REP TILs) was tested against separate sets of autologous tumor cells (TCLs, TCLs + IFNγ or FTDs) and only the highest value reported. T cells were considered reactive if positive for at least one of TNF, IFNγ, or CD107a, minus control.

Although it is generally accepted that tumor types with a higher average TMB respond more frequently to immunotherapy [33,34], the value of TMB for predicting clinical outcome following immunotherapy within tumor types or subtypes is debated and several studies have failed to link TMB to immunological quantitative biomarkers in the TME [5,6,7,9]. On average, TMB is very high in melanoma, but the wide range observed highlights a high heterogeneity across patients [35]. Hence, we examined whether the high inter-sample variability in the proportion of tumor-reactive TILs across melanoma could be explained by the TMB of individual samples. Here, analysis of a smaller melanoma cohort (*n* = 36) did not show any obvious correlation between the proportion of tumor-reactive CD8^+^ or CD4^+^ TILs and TMB (Figure 3, data with TCL/TCL + IFNγ only are shown in Figure S9).

## 3. Discussion

Here, we have presented a functional qualitative analysis of tumor-specific immune responses of TILs across multiple tumor types. Importantly, the proportion of tumor-specific T cells with functional capacity may define the quality of a TIL population, and although multiple parameters associated with an immunologically active TME have been positively associated with response to cancer immunotherapy [5,6,8,36] the predictive value of TIL quality is yet to be established in larger datasets. Early results have shown that, at least for adoptive cell therapy with unselected TILs, a parameter of TIL quality (i.e., defining the amount of tumor-reactive cells infused) may help identifying those patients with a higher likelihood of achieving tumor regression [37,38].

Recent studies have demonstrated that bystander T cells may represent the majority of infiltrating lymphocytes in cancer [10,11]. Here, we quantified the proportion of tumor-reactive T cells across multiple cancer types, and observed that although tumor-reactive CD8^+^ and CD4^+^ T cells could be identified in most samples across cohorts, the proportion was on average remarkably low (especially for non-MM tumors). The estimated proportion of tumor-reactive T cells in situ appeared on average higher when compared to the in vitro analyses, yet still failed to encompass the entire T cell repertoire for most patients and melanoma TILs were more reactive compared to other tumors. These results signify the need for effective T cell selection-strategies in clinical protocols of adoptive cell transfer, especially in non-melanoma tumors. Along this line, next-gen cellular therapy technologies based on T cell selection and selective expansion of tumor-reactive T cells may provide a solution for tumors with low natural immunogenicity. In addition, further studies establishing strategies for bona fide identification and stimulation of truly tumor-specific/tumor-reactive T cells in situ are highly warranted. These novel strategies should focus on avoiding stimulation of deleterious immune sub-populations, such as regulatory T cells, which may account for a fraction of TILs.

For decades, melanoma and RCC have been considered highly immunogenic tumors. This theory is largely supported by multiple reports of occasional spontaneous regression and durable, although minimal, responses to IL-2 [39]. Interestingly, in this study melanomas were on average infiltrated by a greater proportion of CD8^+^ tumor-reactive TILs than other tumor types, yet melanoma PD-1 naïve and PD-1 resistant samples (collected after progression to anti-PD-1) were indistinguishable. This suggests that high infiltration with tumor-reactive CD8^+^ TILs does not appear to guarantee a response to immune checkpoint inhibitors. Additionally, the increased infiltration of tumor-reactive CD8^+^ TILs could not be explained by the differences that we observed in TCR richness or APM machinery activity when comparing melanoma to other tumor types. 

Our analysis of polyfunctionality in vitro revealed differences in TIL-responses across tumors. Distinct functional profiles, albeit without dramatic differences, were detected for CD8^+^ TILs, whereas profiling of CD4^+^ TILs resulted in largely overlapping results across tumor types. At present, the impact of T cell response polyfunctionality on clinical parameters is largely unknown, but the absence of major differences across tumor types indicates that interventions to improve the (low) proportion of tumor-reactive T cell responses may represent a more urgent issue.

Most current data support a model where tumors with high TMB are endowed with a greater number of potential T cell targets (neo-antigens) [2] and are therefore more easily recognized by the immune system. However, TMB does not appear to be well correlated to quantitative biomarkers such as immune infiltration or immune activity in situ [5,6,9]. This is somewhat paradoxical, as both TMB and gene-signatures identifying tumors with high immune infiltration/immune activity in situ can identify patients with a higher likelihood to respond to immunotherapy with checkpoint inhibitors [5,6]. In this study, we expand on these associations by correlating TMB and T cell infiltrate quality (measured as the proportion of tumor-reactive T cells amongst all TILs). Here, although melanoma (the tumor with the highest average TMB) presented with the greatest CD8^+^ TIL response, within melanoma a high TMB did not appear to represent a major driver for the accumulation of either tumor-reactive CD8^+^ TILs or CD4^+^ TILs. Hence, the biological pathways leading to high infiltration by tumor-reactive CD8^+^ TILs in melanomas appears to be independent of TMB. These data confirm that TMB does not strongly correlate to any known immunological parameters across samples, and therefore still functions as a largely immune-independent biomarker. Of note, the major driving forces governing the accumulation of CD8^+^ or CD4^+^ tumor-reactive TILs in the TME are yet to be identified. In our study, we could not find a strong correlation linking the proportion of tumor-reactive CD8^+^ and CD4^+^ TILs in each sample. These data suggest that the accumulation of CD8^+^ or CD4^+^ TIL responses in the TME may follow non-overlapping biological pathways that may not be simultaneously present in an individual tumor or influenced by each other.

This study has some caveats, primarily the low coverage of tumor types (only four in vitro), differences between metastatic or primary tumor sites in distinct tumor types (similar to the composition of samples contained in The Cancer Genome Atlas for melanoma, RCC and SAR), limited number of samples for each of the non-MM tumors in situ and the heterogeneity of in vitro testing due to the extended timeframe (a decade) of sample acquisition and analysis. It was not possible to further characterize regulatory T cells among CD4^+^ TILs, as the TILs used in our in vitro experiments were stimulated with IL-2, hence the expression of FOXP3 was induced in conventional T cells [40]. In addition, although antitumor reactivity testing with autologous cell lines in vitro still represents a gold-standard, the success rate of cell line establishment is variable across tumor types, thereby potentially resulting in a degree of sample selection bias. However, all samples used for in vitro analyses were acquired and analyzed at the same center, guaranteeing a reliable level of consistency. We observed an average higher apparent proportion of tumor-reactive T cells observed in situ compared to in vitro. Due to technical constraints, we used distinct markers to determine the proportion of tumor-reactive T cells in vitro and in situ; in addition, in vitro culturing may influence the clonal composition of TIL preparations, with depletion of tumor-reactive TIL clones because of poor proliferative capacity of dysfunctional cells. These factors may partially (but probably not fully) explain the higher apparent proportion of tumor-reactive T cells observed in situ.

## 4. Materials and Methods 

### 4.1. Patients and Samples

Fresh tumor specimens were obtained via surgical resection or needle biopsy from patients with solid tumors over a ten-year period at the National Center for Cancer Immune Therapy, Copenhagen University Hospital, Herlev, Denmark. Samples were obtained via biopsy collection for enrolment in 11 clinical trials conducted between 2009 and 2020. Written informed consent was provided by all patients prior to obtaining any samples. All trials (NCT00937625, NCT02379195, NCT02354690, H-18055660, NCT02926053, NCT02482090, NCT03287674, NCT03296137, H-4-2012-118, H-15007073, H-2-2014-055) were approved by the relevant Ethics Committee and conducted in accordance with the Declaration of Helsinki and Good Clinical Practice. The clinical cohorts used in this study partially overlap with those that we previously published in other studies [18,19,20,21,22,41,42,43]

Two tumor types (MM and RCC) were selected for their known high immunogenicity and reported sensitivity to immunotherapy; two other tumor types (OC and SAR) were selected for their known relative resistance to checkpoint immunotherapy. All MM and OC originated from metastases, whereas SAR and the majority of RCC originated from primaries. To account for the potential biological differences of tumor samples recovered after progression to anti-PD-1 therapy, MM samples were sub-grouped as anti-PD-1 naïve (MM PD-1 naïve, not previously treated with ant-PD-1 regardless of response to any immunotherapy given after tumor collection) or anti-PD-1 resistant (MM PD-1 res). Overall, these four tumor types are representative of tumors with high (MM), intermediate (RCC and OC), and low (SAR) TMB, according to Yarchoan et al. [9]. 

### 4.2. Establishment of TILs, TCLs, and FTDs

TIL cultures were established in vitro with a two-step process; the initial expansion to generate “Young” or “minimally-cultured” TILs and the REP to generate REP TILs, as previously described in detail [18,19,20,21,22,41]. Short-term autologous TCLs (<10 in vitro passages) were established as described elsewhere using fragments or transport media following scalpel dissection from the same tumor lesion from which the TILs were generated [18,19,20,21,22,41]. All cell lines were generated internally and primarily authenticated via morphology (light microscopy) and in vitro patterns of growth. When in doubt, expression of lineage antigens by PCR or cytospin followed by morphologic evaluation (according to standard cytologic criteria of malignancy [44]) and immunohistochemistry staining of formalin-fixed, paraffin-embedded tissue was carried out. Mycoplasma testing was not performed for all samples. FTDs were obtained from tumor fragments via overnight digestion followed by immediate cryopreservation, as previously described in detail [19,20,22]. 

### 4.3. Assessment of TIL Reactivity Against TCLs or FTDs In Vitro

The level of bulk antitumor reactivity of Y TILs or REP TILs was tested in vitro separately against autologous TCLs, autologous TCLs pre-treated with IFNγ (Peprotech, Stockholm, Sweden; to upregulate tumor antigen processing machinery and presentation, as described elsewhere [45] or autologous FTDs. This was achieved by co-culturing effector (TILs) and target (TCLs, TCLs + IFNγ or FTDs) cells, followed by flow-cytometry analysis of three extensively described type 1 immune response activation markers; TNF, IFNγ and CD107a [18,19,20,22]. Antitumor reactivity was defined as the percentage of live CD8^+^ or CD4^+^ T cells staining positive for at least one of TNF, IFNγ and CD107a, minus control (TILs alone). To define the bulk antitumor reactivity in a given sample, only the highest value obtained from Y TILs or REP TILs tested against TCLs, TCLs + IFNγ or FTDs was reported. For the SAR cohort only, effector-target pairs (only TILs vs TCLs) were pre-tested with co-culture followed by IFNγ ELISPOT, as described previously [46,47]. Further testing with flow cytometry, as described in supplementary methods, was conducted only in those samples with suspected or confirmed ELISPOT reactivity. Therefore, the samples tested only in ELISPOT were not evaluable for CD4^+^ reactivity, because we could not exclude that IFNγ pretreatment of tumors (TCLs + IFNγ) would have resulted in CD4^+^ T cell recognition and positive responses by ELISPOT [18]. SAR samples were not included in the analyses of Y TILs versus REP TILs responses due to the limited number of samples tested with both Y and REP TILs. For polyfunctional characterization of tumor-reactive cells, data were primarily analyzed in FlowJo V10 (BD). Analysis and presentation of distributions was performed using Pestle 2.0 (downloaded from https://niaid.github.io/spice/) and Simplified Presentation of Incredibly Complex Evaluations (SPICE) 6.0 (downloaded from https://niaid.github.io/spice/) according to manufacturer’s instructions. Detailed information can be found in Supplementary Methods. 

### 4.4. TCR Richness and Antigen Processing and Presentation Machinery (APM).

TCR richness and APM machinery were quantified in >1000 samples obtained from TCGA. A detailed description is provided in Supplementary Methods.

### 4.5. Processing of Single-Cell RNA-Sequencing Datasets 

The literature was screened for single-cell RNA-sequencing datasets of tumor biopsies containing data on T cells from individual patients. Eight independent datasets containing single-cell RNA-sequencing data, from 101 tumor biopsies (93 patients) and covering six tumor types (2 breast [28], 14 non-small cell lung [29], 6 hepatocellular [30], 4 renal [31], 8 colorectal [32] cancer and 67 melanoma [25,26,27], were obtained from public repositories or requested directly from the authors and selected for inclusion in our study (Table S4). Only CD8^+^ and CD4^+^ T cells isolated from tumor tissues were utilized. Detailed information can be found in Supplementary Methods, Figure S5A and Figure S10.

### 4.6. Assessment of TIL Reactivity Against Tumor Cells In Situ

Antitumor reactivity within the CD8^+^ and CD4^+^ T cell compartment was defined as the expression of at least one of *TNF, IFNG,* and *TNFRSF9*. Here, although TNF and IFNγ were also used in vitro in all samples due to their high signal-to-noise ratio in activated TILs (barely detectable in TILs alone —significantly upregulated in a variable proportion of TILs recognizing autologous tumor cells), CD137 (encoded by the gene *TNFRSF9*) upregulation was not included in the in vitro analyses. Indeed, the samples used in the study were obtained over a decade, whereas we only recently began to use CD137 as additional tumor-specific (but function-agnostic) T cell activation marker, and confirmed that a proportion accounting for ~20% of the total CD8^+^ tumor-reactive repertoire may be identified by expression of CD137, but not TNF, IFNγ or CD107a [48]. As CD137 may be constitutively expressed on CD4^+^ regulatory T cells [49], we carried out additional analyses on intratumor CD4^+^ T cells using *TNF* and *IFNG* only (Figure S5). We determined that the expression of *LAMP1* (coding for CD107a or lysosome-associated membrane protein-1) could not be used as a T cell degranulation marker in the transcriptomic setting, as its function as part of pre-formed lytic granules in the T cell cytoplasm that are mobilized upon T cell activation requires constitutive mRNA expression, regardless of activation status [50]. In an additional study we could not detect significant upregulation of *LAMP1* on tumor-reactive T cells upon target-recognition (Draghi A et al., in preparation). Therefore, we did not expect *LAMP1* upregulation to be associated with T cell activation/degranulation. Other molecules, such as Granzyme-B (*GZMB*), were expressed in a significant proportion of resting T cells in vitro, and we could therefore not consider these molecules (and their relative mRNA) as bona fide markers of T cell activation. 

### 4.7. Analysis of Tumor Mutational Burden 

Thirty-six samples obtained from patients with melanoma enrolled in interventional clinical trials at the host institution had DNA sequencing data available. Whole-exome sequencing was carried out as previously described [51], and total TMB was calculated based on the somatic non-synonymous single-nucleotide variants detected.

### 4.8. Statistical Analyses

Statistical analyses were carried out using GraphPad Prism 8.4 or SPICE 6.0. Values below 0.5% derived from the subtraction of unstimulated samples from stimulated samples were converted to 0.5% for statistical purposes and generation of figures. All values were expressed as mean unless otherwise specified. The D’Agostino and Pearson normality test was performed to determine whether the data were normally distributed. Mann–Whitney or Wilcoxon-matched pairs tests were used to determine statistical significance in case of non-normally distributed data. Unpaired or paired T tests were used to determine statistical significance in case of normally distributed data. Correlations were expressed by Spearman and Pearson R value in case of non-normally and normally distributed data, respectively. Regarding statistical analyses on TCGA data, for pairwise comparisons of multiple groups, with non-equal variances and samples sizes, the non-parametric Games–Howell post-hoc test was used. Benjamini Hochberg method was selected as the adjustment method for *p*-values for multiple comparisons. Significance level for *p*-adjusted values was set to 0.05.

## 5. Conclusions

In conclusion, multiple studies have shown that exploiting the T cell-infiltrates of solid tumors has great therapeutic potential across tumor types [22,37,52,53,54,55]. However, a large proportion of tumor-infiltrating T cells, especially in TIL cultures obtained from non-MM tumors, are not tumor-relevant. These data indicate that future strategies employing immunotherapies based on T cell infusion or stimulation of T cells residing in the TME should be tailored to the tumor-reactive T cell subpopulation. Parameters such as TMB, which are related to the overall burden of neo-antigens, may not be relevant to address these issues.

## Supplementary Materials

The following are available online at https://www.mdpi.com/2072-6694/12/11/3344/s1, Figure S1: Comparison of multiple sources of tumor targets and TILs (in vitro), Figure S2: Antitumor-reactivity of TILs across clinical cohorts (in vitro, TCL/TCL+IFNγ only), Figure S3: TCR richness and antigen processing and presentation machinery across four tumor types, Figure S4: Antitumor-reactivity of CD8+ and CD4+ TILs across tumor types (in vitro, pooled data), Figure S5: Proportion of tumor-reactive CD4+ TILs calculated using two markers, TNF and IFNG (scRNAseq in situ), Figure S6: Polyfunctional characterization of CD8+ tumor-reactive TILs in MM (in vitro, functions combination), Figure S7: Polyfunctional characterization of CD8+ tumor-reactive TILs across tumor types (in vitro, functions combination), Figure S8: Polyfunctional characterization of CD4+ tumor-reactive TILs across tumor types (in vitro, functions combination), Figure S9: Antitumor-reactivity of TILs and tumor mutational burden in melanoma (in vitro, TCL/TCL+IFNγ only), Figure S10: Proportion of tumor-reactive TILs in PD1-naïve and PD-1 res samples (scRNAseq in situ), Table S1: Overview of all samples used in the study (in vitro data), Table S2: Overview of CD8+ TILs analyses (in vitro data), Table S3: Overview of CD4+ TILs analyses (in vitro data), Table S4: Single-cell RNA-sequencing datasets accession numbers (in situ data).
