# Supplementary material for: Qualitative Analysis of Tumor-Infiltrating Lymphocytes across Human Tumor Types Reveals a Higher Proportion of Bystander CD8+ T Cells in Non-Melanoma Cancers Compared to Melanoma
Source: Cancers (Basel). 2020 Nov 12;12(11):3344. doi: 10.3390/cancers12113344 (PMC7696049; doi:10.3390/cancers12113344)
Supplement: Supplementary file 1 [file cancers-12-03344-s001.pdf]

Supplementary Materials

Qualitative Analysis of Tumor-Infiltrating Lymphocytes across Human Tumor Types Reveals a Higher Proportion of Bystander CD8⁺ T Cells in Non-Melanoma Cancers Compared to Melanoma

Aishwarya Gokuldass, Arianna Draghi, Krisztian Papp, Troels Holz Borch, Morten Nielsen, Marie Christine Wulff Westergaard, Rikke Andersen, Aimilia Schina, Kalijn Fredrike Bol, Christopher Aled Chamberlain, Mario Presti, Özcan Met, Katja Harbst, Martin Lauss, Samuele Soraggi, Istvan Csabai, Zoltan Szallasi, Göran Jönsson, Inge Marie Svane and Marco Donia

Supplementary Figures

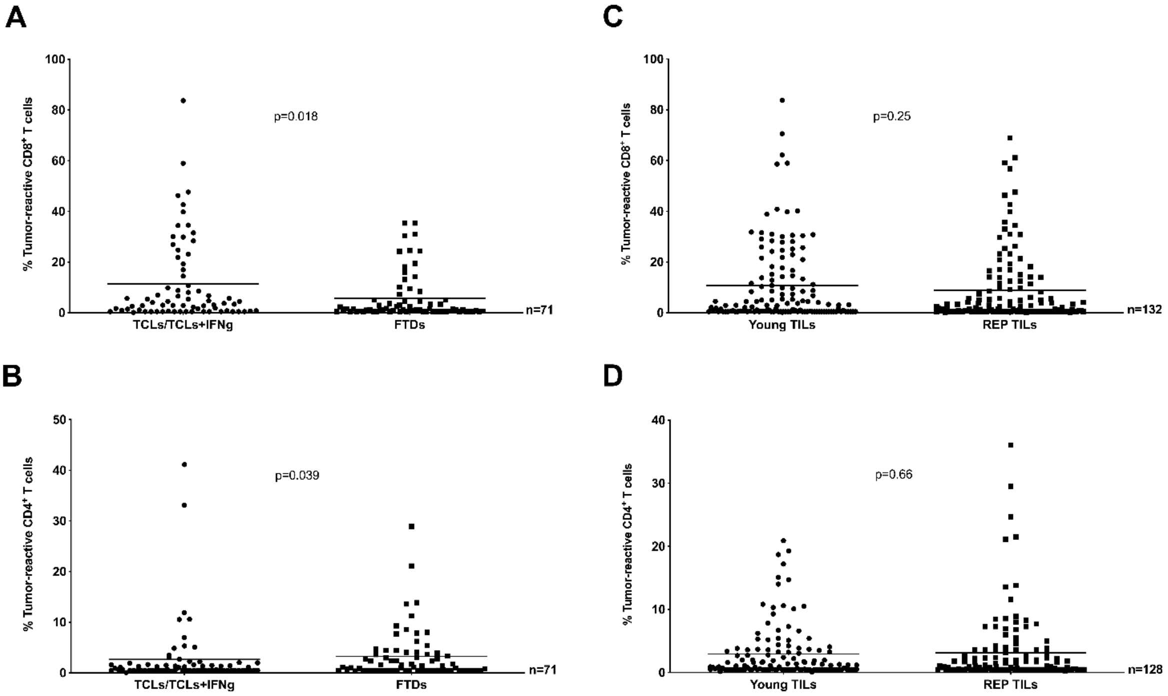

Figure S1. Comparison of multiple sources of tumor targets and TILs (in vitro). (A and B) 71 TILs were tested separately against TCL/TCL+IFNγ and FTDs. Testing against FTDs yielded a lower reactivity for $CD8^+$ (**A**) and a higher reactivity for $CD4^+$ (**B**) TILs. (**C**) 132 tumors (TCLs, TCLs+IFNγ or FTDs) were used as target cells for recognition by $CD8^+$ Y TILs or REP TILs. (**D**) 128 tumors (TCL, TCL+IFNγ and/or FTDs) were used as target cells for recognition by $CD4^+$ Y TILs or REP TILs. No significant differences were observed (C and D). (A–D) Only the highest value of recognition is reported in the figures and T cells were considered reactive if positive for at least one of TNF, IFNγ or CD107a, minus control.

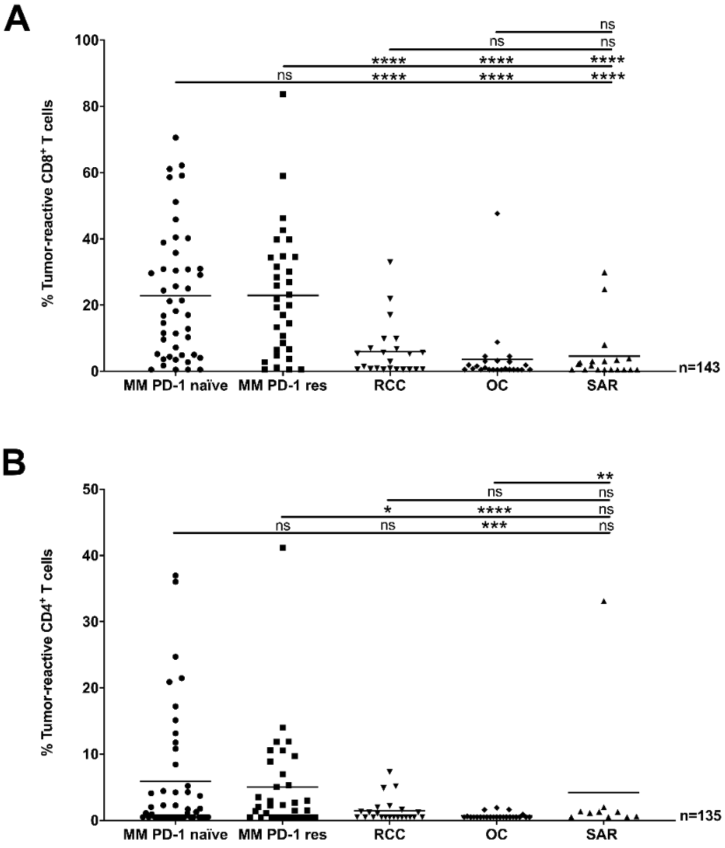

Figure S2. Antitumor-reactivity of TILs across clinical cohorts (in vitro, TCL/TCL+IFNγ only). (**A**) Similar to Figure 1A, the proportion of tumor-reactive CD8+ TILs was significantly greater in MM cohorts compared to other tumor types, with no difference related to previous exposure to anti-PD-1 therapy. (**B**) Some differences in the proportion of tumor-reactive CD4+ TILs could be observed across cohorts, although the MM cohorts had a remarkably higher proportion of reactive CD4+ only in comparison with the OC cohort. (A,B) In these panels, the recognition of TILs (Y TILs and REP TILs) was tested against autologous TCLs or TCLs+IFNγ, and only the highest value reported. T cells were considered reactive if positive for at least one of TNF, IFNγ or CD107a, minus control. Mann-Whitney test, * $p < 0.05$, ** $p < 0.01$, *** $p < 0.001$, **** $p < 0.0001$, ns: no statistical significance.

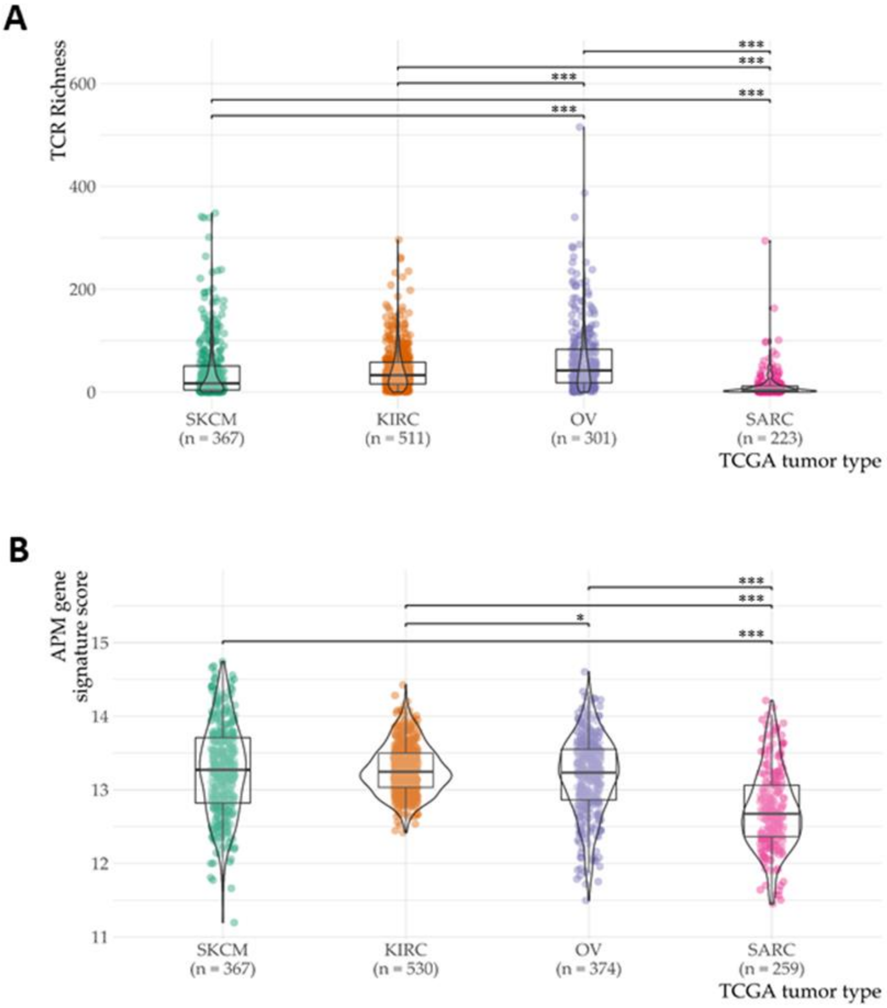

Figure S3. TCR richness and antigen processing and presentation machinery across four tumor types. (**A**) Visualization of the TCR richness distribution across four different tumor types. (**B**) Visualization of the antigen processing and presentation machinery score (calculated based on an APM gene signature as described in Supplementary Methods) across four different tumor types. Data from four TCGA datasets, representing the same tumor types used in the in vitro study (skin cutaneous melanoma (SKCM), kidney renal clear cell carcinoma (KIRC), ovarian serous cystadenocarcinoma (OV), sarcoma (SARC)), were pooled and utilized to generate these box plots. For SKCM, only data from metastatic samples were employed. TCGA study abbreviations are reported at https://gdc.cancer.gov/resources-tcga-users/tcga-code-tables/tcga-study-abbreviations. * $p_{\text{FDR-corrected}} < 0.05$, ** $p_{\text{FDR-corrected}} < 0.01$, *** $p_{\text{FDR-corrected}} < 0.001$ in Games-Howell test. Only significant comparisons are shown.

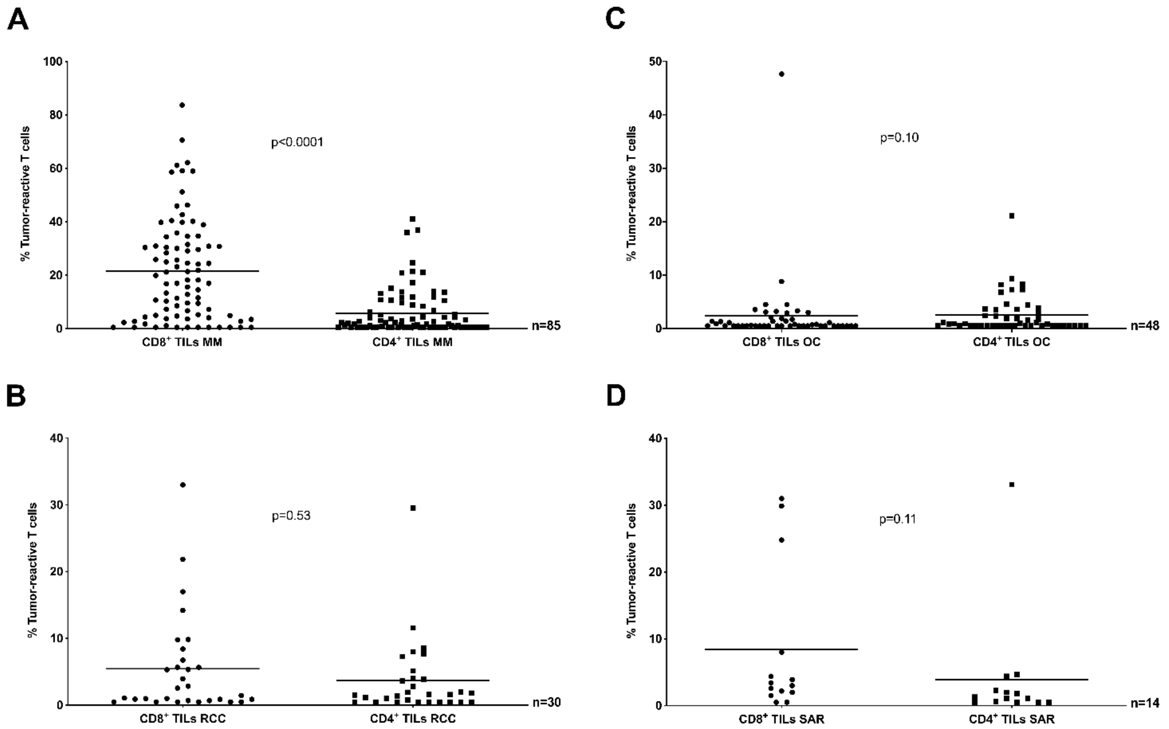

Figure S4. Antitumor-reactivity of CD8⁺ and CD4⁺ TILs across tumor types (in vitro, pooled data). (**A**) The proportion of tumor-reactive CD8⁺ TILs was greater than CD4⁺ TILs in MM cohorts (pooled, similar results obtained segregating PD-1 naïve and PD-1 res). (**B**, **C** and **D**) No significant differences were observed in the other tumor types. (A–D) The recognition of TILs (Y TILs and REP TILs) was tested against separate sets of autologous tumor cells (TCLs, TCLs+IFNγ or FTDs) and only the highest value reported. T cells were considered reactive if positive for at least one of TNF, IFNγ or CD107a, minus control.

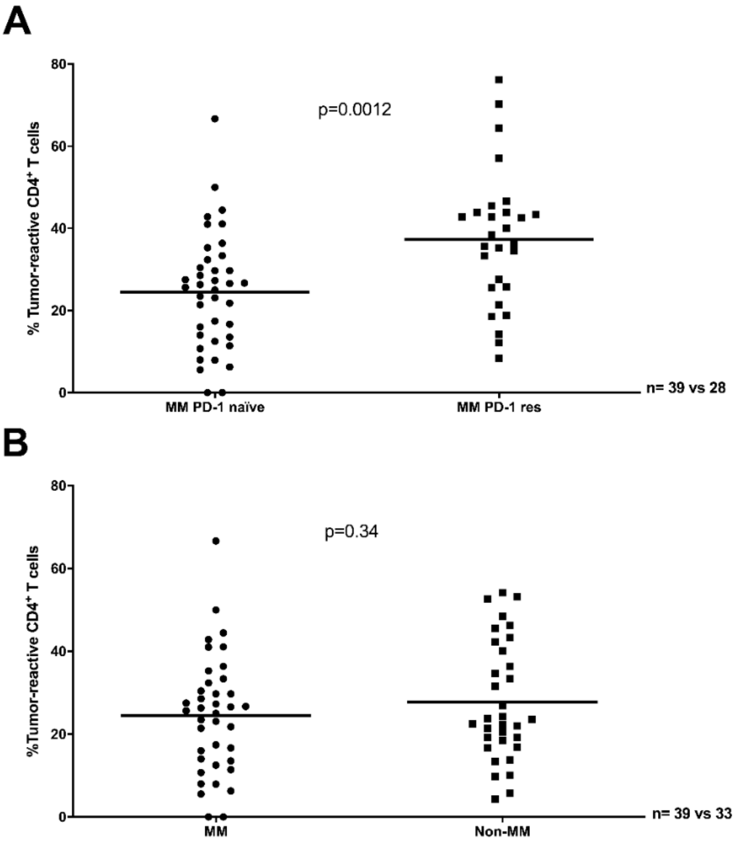

Figure S5. Proportion of tumor-reactive CD4+ TILs calculated using two markers, *TNF* and *IFNG* (scRNAseq in situ). (**A**) The proportion of tumor-reactive CD4+ TILs was higher in MM PD-1 res samples compared to MM PD-1 naïve (Unpaired *t* test, $p = 0.0012$). For the analyses shown in this panel, all MM samples classified as PD-1 naïve ($n = 39$) or PD-1 res ($n = 28$) were utilized (for eight patients, data from both MM PD-1 naïve and MM PD-1 res biopsies were available). (**B**) The proportion of tumor-reactive CD4+ TILs was similar in MM (PD-1 naïve only) compared to non-MM (Unpaired *t* test, $p = 0.34$). (A,B) T cells were considered reactive if positive for the expression of either *TNF* or *IFNG*.

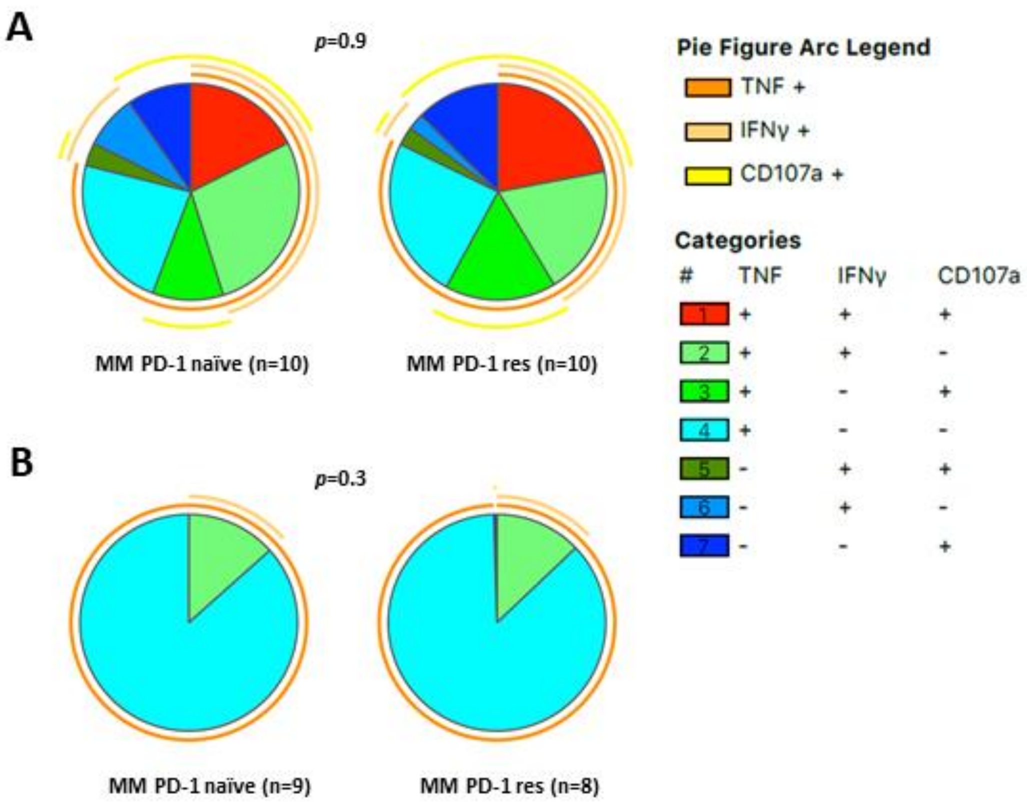

Figure S6. Polyfunctional characterization of CD8+ tumor-reactive TILs in MM (in vitro, functions combination). The pie charts illustrate the relative distribution of the seven combinations of three T cell functions generated by tumor-reactive CD8+ (**A**) and CD4+ (**B**) T cells in two different cohorts: MM PD-1 naïve and MM PD-1 res. The red, green and blue slices represent cells expressing either three, two or one of the three T cell functions analyzed, respectively. CD8+ and CD4+ TILs in the two MM cohorts had a comparable polyfunctionality (Permutation test, $p = 0.9$ and $p = 0.3$, respectively). (A,B) The figure shows a graphical presentation of SPICE data analyses. In these panels, the recognition of Y TILs was tested against TCLs or TCLs+IFNγ, and only the highest value reported. T cells were gated on cells expressing at least one of the three T cell functions analyzed (TNF, IFNγ and CD107a). Median values are shown.

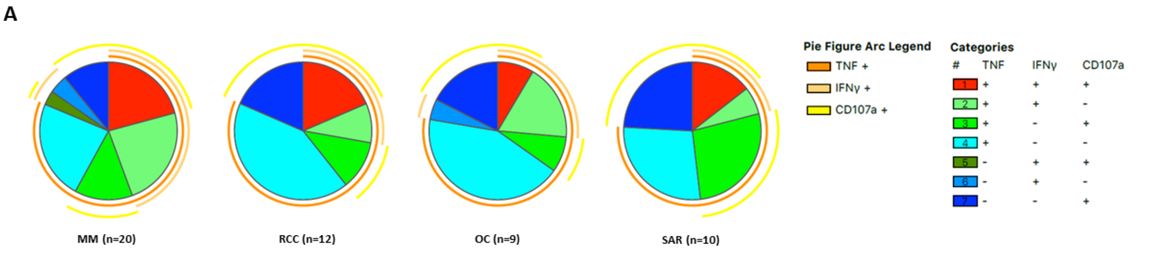

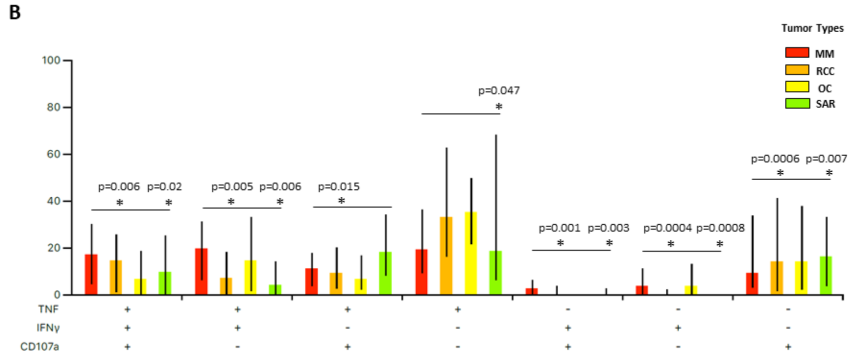

C

Permutation test

	MM	RCC	OC	SAR
MM		0.0294	0.1049	0.0247
RCC	0.0294		0.5865	0.7115
OC	0.1049	0.5865		0.3737
SAR	0.0247	0.7115	0.3737	

Figure S7. Polyfunctional characterization of CD8⁺ tumor-reactive TILs across tumor types (in vitro, functions combination). (**A**) The pie charts and (**B**) the bar charts illustrate the relative distribution of the seven combinations of three T cell functions generated by tumor-reactive CD8⁺ T cells within each individual tumor type. Black bars represent standard deviations. In the pie charts, the red, green and blue slices represent cells expressing either three, two or one of the three T cell functions analyzed, respectively. (**C**) The table shows the results of the Permutation Test performed between the different tumor types. Significant values (<0.05) are highlighted in red. The polyfunctional characterization of tumor-reactive CD8⁺ T cells in RCC and SAR was significantly different from MM. No statistically significant differences were observed between all other tumor types. (A–C) The figure shows a graphical presentation of SPICE data analyses. In these panels, the recognition of Y TILs was tested against TCLs or TCLs+IFNγ, and only the highest value reported. T cells were gated on cells expressing at least one of the three T cell functions analyzed (TNF, IFNγ and CD107a). Median values are shown. * $p < 0.05$ in Wilcoxon Rank Sum Test.

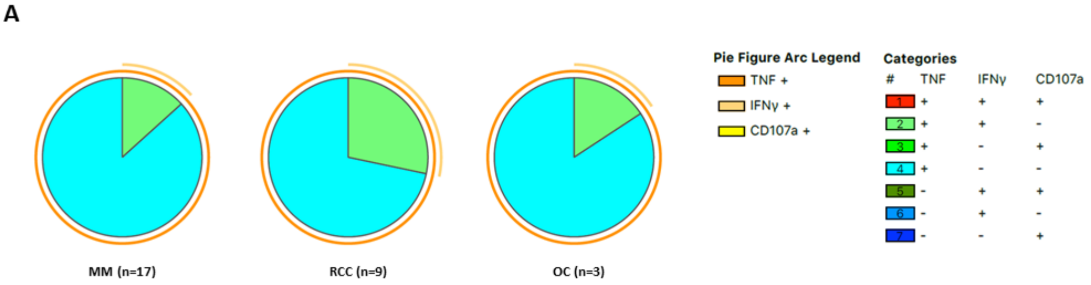

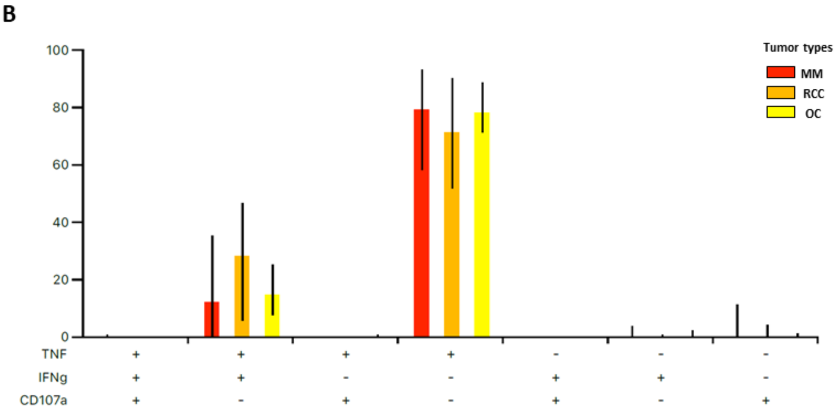

C

Permutation test

	MM	RCC	OC
MM		0.4082	0.8314
RCC	0.4082		0.4959
OC	0.8314	0.4959	

Figure S8. Polyfunctional characterization of CD4⁺ tumor-reactive TILs across tumor types (in vitro, functions combination). (**A**) The pie charts and (**B**) the bar charts show the relative distribution of the seven combinations of the three T cell functions expressed by tumor-reactive CD4⁺ T cells within each individual tumor type. Black bars represent standard deviations. In the pie charts, the green and blue slices represent cells expressing either two or one of the three T cell functions analyzed, respectively. (**C**) The table shows the results of the Permutation Test performed between the different tumor types. No statistically significant differences were observed between the three tumor types. (A–C) The figure shows a graphical presentation of SPICE data analyses. In these panels, the recognition of Y TILs was tested against TCLs or TCLs+IFNγ, and only the highest value reported. T cells were gated on cells expressing at least one of the three T cell functions analyzed (TNF, IFNγ and CD107a). Median values are shown.

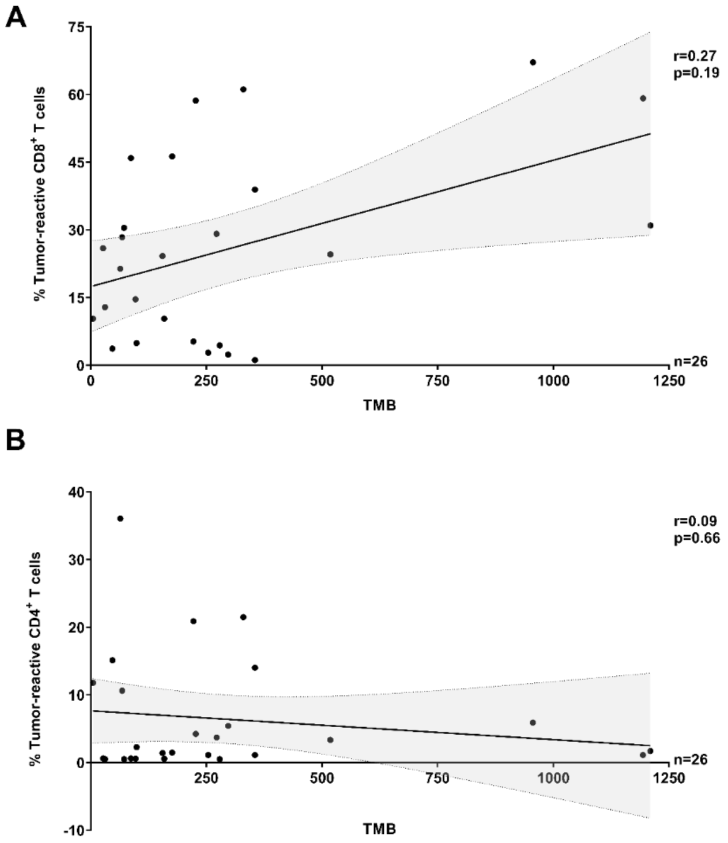

Figure S9. Antitumor-reactivity of TILs and tumor mutational burden in melanoma (in vitro, TCL/TCL+IFNγ only). The proportion of tumor-reactive (**A**) $CD8^+$ and (**B**) $CD4^+$ TILs was not correlated (Spearman $r = 0.27$, $p = 0.19$ and Spearman $r = 0.09$, $p = 0.66$, respectively) to TMB (pooled MM clinical cohorts). The solid lines and dotted lines represent the best-fit regression line and 95% confidence interval, respectively. In these panels, the recognition of TILs (Y TILs and REP TILs) was tested against autologous TCLs or TCLs+IFNγ, and only the highest value reported. T cells were considered reactive if positive for at least one of TNF, IFNγ or CD107a, minus control.

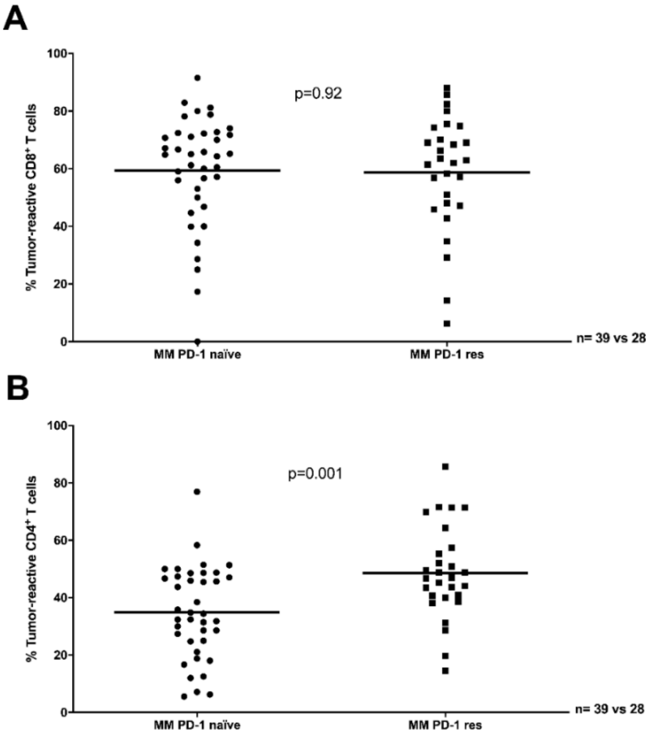

Figure S10. Proportion of tumor-reactive TILs in PD1-naïve and PD-1 res samples (scRNAseq in situ). (**A**) The proportion of tumor-reactive CD8⁺ TILs was not significantly different in MM PD-1 naïve compared to MM PD-1 res samples (Mann-Whitney test, p = 0.92). (**B**) The proportion of tumor-reactive CD4⁺ TILs was higher in MM PD-1 res compared to MM PD-1 naïve samples (Unpaired t test, p = 0.001). (A–B) T cells were considered reactive if positive for the expression of least one of *TNF*, *IFNG*, or *TNFRSF9*. For the analyses shown in these panels, all MM samples classified as PD-1 naïve (n = 39) or PD-1 res (n = 28) were utilized (for eight patients, data from both MM PD-1 naïve and MM PD-1 res biopsies were available).

Supplementary Tables

Table S1. Overview of all samples used in the study (in vitro data).

Clinical Cohort	Overall			
	Total Number of Samples	CD8+ and CD4+ TIL Data Available	Only CD8+ TIL Data Available	Only CD4+ TIL Data Available
MM PD-1-naïve	46	46	0	0
MM PD-1-res	40	39	0	1
RCC	30	30	0	0
OC	48	48	0	0
SAR	23	14	9	0
**All**	**187**	**177**	9	1

Table S2. Overview of CD8+ TILs analyses (in vitro data).

Clinical Cohort	CD8+ TILs						
	CD8+ TIL Data Available	Samples Tested with TCL and FTD	Samples Tested with TCL Only	Samples Tested with FTD Only	Samples Tested with Y and REP TILs	Samples Tested with Y TILs Only	Samples Tested with REP TILs Only
MM PD-1-naïve	46	1	44	1	29	2	15
MM PD-1-res	39	21	11	7	30	6	3
RCC	30	19	4	7	28	0	2
OC	48	21	3	24	45	1	2
SAR	23	9	10	4	9	10	4
**All**	**186**	**71**	72	43	141(**132***)	19	26

*Actual value used when testing for potential differences in reactivity among pairs tested separately with Y TILs and REP TILs.

Table S3: Overview of $CD4^+$ TILs analyses (in vitro data)

Clinical Cohort	CD4⁺ TILs						
	$CD4^+$ TIL Data Available	Samples Tested with TCL and FTD	Samples Tested with TCL Only	Samples Tested with FTD Only	Samples Tested with Y and REP TILs	Samples Tested with Y TILs Only	Samples Tested with REP TILs Only
MM PD-1-naïve	46	1	44	1	25	2	19
MM PD-1-res	40	21	12	7	30	6	4
RCC	30	19	4	7	28	0	2
OC	48	21	3	24	45	1	2
SAR	14	9	1	4	9	1	4
**All**	**178**	**71**	64	43	137(**128***)	10	31

*Actual value used when testing for potential differences in reactivity among pairs tested separately with Y TILs and REP TILs.

Table S4. Single-cell RNA-sequencing datasets accession numbers (in situ data).

Dataset	GEO Accession	Other Source
Savas et al. [1]	GSE110686	
Guo et al. [2]	GSE99254	
Zheng et al [3]	GSE98638	
Young et al. [4]		"Additional Data S1" from Reference [4]
Zhang et al. [5]	GSE108989	
Tirosh et al. [6]	GSE115978	
Jerby-Arnon et al. [7]	GSE115978	
Sade-Feldman et al. [8]		Obtained directly from authors

Supplementary Methods

Assessment of TIL Reactivity Against TCLs or FTDs in Vitro

Autologous TILs were thawed and rested overnight in RPMI 1640 (Cat. No 72400-021, Gibco, Thermo Fisher Scientific, Waltham, MA, USA) containing 10% Human serum (H4522, Sigma-Aldrich/Merck KGaA, Darmstadt, Germany) and 1% PenStrep (Cat No 15140122, Gibco, Thermo Fisher Scientific). For the analysis of TIL reactivity against FTDs, single-cell suspensions were thawed, washed and used immediately after a trypan blue viability count. Tumor-specific immune activation was assessed with a 5 to 8-hour co-culture assay of TILs and autologous TCLs or FTDs (effector/target ratio of 3:1) in the presence of anti-CD107a antibody and Brefeldin A (dilution of 1:1000, GolgiPlug™, Cat No 555029, BD Biosciences, San Jose, CA, USA) or a combination of Brefeldin A and Monensin (dilution of 1:1000, GolgiStop™, Cat No 554724, BD). Intracellular staining of TILs and acquisition with a flow cytometer were carried out using standard methods. Boolean gating was performed to obtain the proportion of cells positive for at least one marker among TNF, IFNγ and CD107a. A specific antitumor response was defined as the detection of a response greater than twice the background (i.e., TILs alone), with a difference of > 0.5% from the background and a minimum of 50 positive flow cytometry events after subtraction of the control. A value of 0.5% was used as the limit of sensitivity. Unspecific T cell activation was ruled out by co-culturing selected TILs with a panel of allogeneic melanoma TCLs in our institution's cell line bank, with no upregulation of functional markers detected when co-culturing with at least one TCL.

Antibodies

The following antibodies were used for the flow cytometry assays: anti-CD3 (FITC, clone SK7, Cat No 345764, BD), anti-CD56 (PE, clone NCAM16.2, Cat No 345812, BD), anti-CD3 (PE-CF594, clone UCHT1, Cat No 562280, BD), anti-CD4 (PerCP, clone OKT4, Cat No 317432, BioLegend, San Diego, CA, USA), anti-CD8 (PerCP, clone SK1, Cat No 345774, BD), anti-CD4 (PerCP-Cy5.5, BD), anti-IFNγ (PE-Cy7, clone B27, Cat No 557643, BD), anti-TNF (APC, clone MAb11, Cat No 554514, BD), Live/Dead Fixable Dead Cell Stain Near-IR NIR (APC-Cy7, Cat No L34976, Thermo Fisher), anti-CD107a (BV421, clone H4A3, Cat No 562623, BD), anti-CD4 (BV510, clone SK3, Cat No 562970, BD), anti-CD56 (BV510, clone NCAM16.2, Cat No 563041, BD), anti-CD8 (Qdot605, clone 3B5, Cat No Q10009, Thermo Fisher), anti-CD4 (BV711, clone SK3, Cat No 563028, BD).

Characterization of TIL Polyfunctionality

For polyfunctional characterization of tumor-reactive TILs, flow data were primarily analyzed in FlowJo V10 (BD). Lymphocytes were selected based on a plot of FSC-A vs SSC-A. Doublets were removed by gating FSC-A vs FSC-H. Subsequently, cells negative for Live/Dead Fixable Dead Cell Stain Near-IR (NIR) were gated as live cells and $CD3^+$ TILs were gated in a plot of CD3 vs CD56. $CD4^+$ and $CD8^+$ populations were identified in a CD4 vs CD8 plot. Finally, TNF^+, $IFN\gamma^+$ and $CD107a^+$ subpopulations, within the CD4 and CD8 compartments, were gated in a plot of TNF or IFNγ or CD107a versus CD4 or CD8, respectively. Subsequently, Boolean combination gates were made for the three functional markers (TNF, IFNγ, and CD107a), generating seven gates of tumor-reactive TILs, each showing the percentage of $CD8^+$ or $CD4^+$ TILs expressing a unique combination of the three markers ($TNF^+/IFN\gamma^+/CD107a^+$, $TNF^+/IFN\gamma^+/CD107a^-$, $TNF^+/IFN\gamma^-/CD107a^+$, $TNF^-/IFN\gamma^+/CD107a^+$, $TNF^+/IFN\gamma^-/CD107a^-$, $TNF^-/IFN\gamma^+/CD107a^-$, $TNF^-/IFN\gamma^-/CD107a^+$) and one gate of not reactive TILs ($TNF^-/IFN\gamma^-/CD107a^-$). Only the data regarding the seven gates of tumor-reactive TILs were used for the following analyses and exported into Pestle 2.0, where they were formatted according to manufacturer's instructions, and the background was subtracted. In SPICE, thresholds were set at 0.1. Comparison of pie charts was performed using a partial permutation test followed by comparison of bar charts using a Wilcoxon rank sum test in case of significant difference between the respective pies, as described previously [9]. All values calculated in SPICE were expressed as median unless otherwise specified. Three to 12 samples were selected for these analyses from each tumor type

according to the following criteria: 1) Only samples with a proportion of tumor-reactive T cells > 0.5% were selected; 2) For each reported patient, only the sample with highest reactivity obtained from Y TILs tested against TCLs or TCLs + IFNγ was utilized; 3) Within each tumor type, samples were selected along all the range of reactivity, with an equal representation of samples with high (>66 percentile), intermediate (33–66 percentile) or low (<33 percentile) reactivity.

Processing of TCGA Data

The Cancer Genome Atlas (TCGA) sample information were downloaded via the TCGAbiolinks R package [10–12] . Cancer types data were retrieved via the TCGAbiolinks R package to generate the sub-categories of Skin cutaneous melanoma (SKCM), Kidney renal clear cell carcinoma (KIRC), Ovarian serous cystadenocarcinoma (OV) and Sarcoma (SARC). TCGA study abbreviations used in this study are reported at https://gdc.cancer.gov/resources-tcga-users/tcga-code-tables/tcga-study-abbreviations. The samples information table was filtered to remove Formalin-Fixed Paraffin-Embedded (FFPE) samples and replicates. Samples labeled as Metastatic (for SKCM) or as Primary Solid Tumor (for KIRC and SARC) or any tumor (for OV) and as RNA-Seq were selected. The TCGA HTSeq-Counts RNA-Seq data were downloaded using the TCGA biolinks R package and normalized using the DESeq2 R package, with the variance stabilizing transformation vst() function. A gene list of 17 genes (*B2M, TAP1, TAPBPL, CALR, PSMB9, PSMB10, ERAP1, PDIA3, NLRC5, RFX5, PSME1, PSME2, PSME3, CIITA, HSP90AB1, HSP90AA1,* and *HSP90B1*) that reflected antigen processing and presentation machinery (APM) was obtained from Thompson et al. [13].The vst-normalized count data were used for the calculation of an APM score based on the mean normalized expression of the above mentioned 17 genes for each selected sample. TCR richness data for the selected samples were obtained from Thorsson et al. [14](Table S1) and calculated from RNA-seq datasets as previously described [14]. Plots and statistical tests for the TCGA data were produced using the ggstatsplot R package[15].

T cell Transcriptomics Single-Cell Data from Public Domain

One dataset [16] was downloaded but not used for further analysis due to a significantly lower average number of detected genes per cell. Of the twelve colorectal cancer patients from Zhang et al [5], four were excluded due to the microsatellite unstable (MSI) status of their disease. The exceedingly high TMB of these samples could bias the analysis due to the high disproportionality of tumor-reactive T cells (this condition is normally found in only 15% of all patients with colorectal cancer [17]. Therefore, only microsatellite stable (MSS) colorectal cancer samples were considered for the analyses in this study. Additionally, only 4 tumor samples from adult patients (1 papillary renal cell carcinoma and 3 clear cell renal cell carcinomas) were included from a kidney cancer dataset [18] . The melanoma datasets [6–8] included samples from both anti-PD-1 therapy naïve and previously-treated patients. Treatment with anti-PD-1 is known to induce early infiltration of intratumoral T cell, especially in connection with an objective response [19], however we did not include any tumor biopsy from regressing lesions under treatment with anti-PD-1 in the in vitro study. Hence, samples from patients whose lesions regressed after anti-PD-1 therapy were excluded from the in situ analyses to maintain consistency with our in vitro data. The remaining samples (n = 67) were classified as MM PD-1 naïve (n = 39) or MM PD-1 res (n = 28). Both MM PD-1 naïve and MM PD-1 res biopsies were available for eight patients, but only the biopsies collected at the earlier time point (MM PD-1 naïve) were considered for these analyses (in total 59 samples from an equal number of individual patients). For those patients where multiple MM PD-1 res biopsies were available, the one collected at the latest time point was chosen. For $CD4^+$ TILs, only samples from MM PD-1 naïve patients (n = 39) were considered (the proportion of tumor-reactive TILs was significantly higher in MM PD-1-res samples, therefore the two groups could not be merged), whereas both MM PD-1 naïve and MM PD-1 res samples (n = 59) were considered for $CD8^+$ TILs (no difference in the proportion of tumor-reactive TILs between MM PD-1 naïve and MM PD-1 res samples) (Figure S5A and Figure S10). For patient Mel129 from Jerby-Arnon et al [7], data from two biopsies collected at the same time point were available, and the mean values of the two were used. Patient Mel78 from Tirosh et al [6] was excluded

from the analyses due to the extremely low number of T cells isolated from the tumor tissue, whereas, no $CD4^+$ T cells could be identified from patient P1207 from Zhang et al. after data processing [5]. As different gene IDs were used by the various authors who published these datasets, we used the HUGO Gene Nomenclature Committee (https://www.genenames.org/download/custom/, accessed on 21/11/2019) to convert gene IDs to a NCBI GeneID in each dataset. Only T cells isolated from tumor tissues were utilized. Additionally, only those genes expressed in at least three cells and only those cells expressing at least 500 genes were considered for these analyses. Normalization of the read counts was performed with the SCTransform function in the Seurat R package. Identification of $CD4^+$ and $CD8^+$ T cells was based on CD4, CD8A and CD8B expression and on the following criteria:

CD4 T cells = CD4+ AND CD8A- AND CD8B-

CD8 T cells = CD4- AND (CD8A+ OR CD8B+)

Cells were considered "positive" or "negative" for the expression of a specific gene according to a manually set threshold based on a bimodal distribution of the gene expression across the different datasets.

References

1. Savas, P.; Virassamy, B.; Ye, C.; Salim, A.; Mintoff, C.P.; Caramia, F.; Salgado, R.; Byrne, D.J.; Teo, Z.L.; Dushyanthen, S.; et al. Single-cell profiling of breast cancer T cells reveals a tissue-resident memory subset associated with improved prognosis. *Nat. Med.* **2018**, *24*, 986–993, doi:10.1038/s41591-018-0078-7.
2. Guo, X.; Zhang, Y.; Zheng, L.; Zheng, C.; Song, J.; Zhang, Q.; Kang, B.; Liu, Z.; Jin, L.; Xing, R.; et al. Global characterization of T cells in non-small-cell lung cancer by single-cell sequencing. *Nat. Med.* **2018**, *24*, 978–985, doi:10.1038/s41591-018-0045-3.
3. Zheng, C.; Zheng, L.; Yoo, J.K.; Guo, H.; Zhang, Y.; Guo, X.; Kang, B.; Hu, R.; Huang, J.Y.; Zhang, Q.; et al. Landscape of Infiltrating T Cells in Liver Cancer Revealed by Single-Cell Sequencing. *Cell* **2017**, *169*, 1342–1356.e16, doi:10.1016/j.cell.2017.05.035.
4. Young, M.D.; Mitchell, T.J.; Vieira Braga, F.A.; Tran, M.G.B.; Stewart, B.J.; Ferdinand, J.R.; Collord, G.; Botting, R.A.; Popescu, D.M.; Loudon, K.W.; et al. Single-cell transcriptomes from human kidneys reveal the cellular identity of renal tumors. *Science (80-.).* **2018**, *361*, 594–599, doi:10.1126/science.aat1699.
5. Zhang, L.; Yu, X.; Zheng, L.; Zhang, Y.; Li, Y.; Fang, Q.; Gao, R.; Kang, B.; Zhang, Q.; Huang, J.Y.; et al. Lineage tracking reveals dynamic relationships of T cells in colorectal cancer. *Nature* **2018**, 1, doi:10.1038/s41586-018-0694-x.
6. Tirosh, I.; Izar, B.; Prakadan, S.M.; Ii, M.H.W.; Treacy, D.; Trombetta, J.J.; Rotem, A.; Rodman, C.; Lian, C.; Murphy, G.; et al. Dissecting the multicellular ecosystem of metastatic melanoma by single-cell RNA-seq. *Science (80-.).* **2016**, *352*, 189–196, doi:10.1126/science.aad0501.Dissecting.
7. Jerby-Arnon, L.; Shah, P.; Cuoco, M.S.; Rodman, C.; Su, M.J.; Melms, J.C.; Leeson, R.; Kanodia, A.; Mei, S.; Lin, J.R.; et al. A Cancer Cell Program Promotes T Cell Exclusion and Resistance to Checkpoint Blockade. *Cell* **2018**, *175*, 984–997.e24, doi:10.1016/j.cell.2018.09.006.
8. Sade-Feldman, M.; Yizhak, K.; Bjorgaard, S.L.; Ray, J.P.; Boer, C.G. de; Jenkins, R.W.; Lieb, D.J.; Chen, J.H.; Frederick, D.T.; Barzily-Rokni, M.; et al. Defining T Cell States Associated with Response to Checkpoint Immunotherapy in Melanoma. *Cell* **2018**, *175*, 998–1013.e20, doi:10.1016/J.CELL.2018.10.038.
9. Roederer, M.; Nozzi, J.L.; Nason, M.C. SPICE: exploration and analysis of post-cytometric complex multivariate datasets. *Cytom. Part A* **2011**, *79*, 167–74, doi:10.1002/cyto.a.21015.
10. Colaprico, A.; Silva, T.C.; Olsen, C.; Garofano, L.; Cava, C.; Garolini, D.; Sabedot, T.S.; Malta, T.M.; Pagnotta, S.M.; Castiglioni, I.; et al. TCGAbiolinks: an R/Bioconductor package for integrative analysis of TCGA data. *Nucleic Acids Res.* **2016**, *44*, e71–e71, doi:10.1093/nar/gkv1507.
11. Silva, T.C.; Colaprico, A.; Olsen, C.; D'Angelo, F.; Bontempi, G.; Ceccarelli, M.; Noushmehr, H. TCGA Workflow: Analyze cancer genomics and epigenomics data using Bioconductor packages. *F1000Research* **2016**, *5*, 1542, doi:10.12688/f1000research.8923.2.
12. Mounir, M.; Lucchetta, M.; Silva, T.C.; Olsen, C.; Bontempi, G.; Chen, X.; Noushmehr, H.; Colaprico, A.; Papaleo, E. New functionalities in the TCGAbiolinks package for the study and integration of cancer data from GDC and GTEx. *PLoS Comput. Biol.* **2019**, *15*, e1006701, doi:10.1371/journal.pcbi.1006701.
13. Thompson, J.C.; Davis, C.; Deshpande, C.; Hwang, W.T.; Jeffries, S.; Huang, A.; Mitchell, T.C.; Langer, C.J.; Albelda, S.M. Gene signature of antigen processing and presentation machinery predicts response to

checkpoint blockade in non-small cell lung cancer (NSCLC) and melanoma. *J. Immunother. Cancer* **2020**, *8*, doi:10.1136/jitc-2020-000974.

14. Thorsson, V.; Gibbs, D.L.; Brown, S.D.; Wolf, D.; Bortone, D.S.; Ou Yang, T.H.; Porta-Pardo, E.; Gao, G.F.; Plaisier, C.L.; Eddy, J.A.; et al. The Immune Landscape of Cancer. *Immunity* **2018**, *48*, 812–830.e14, doi:10.1016/j.immuni.2018.03.023.
15. Patil Indrajeet ggstatsplot: "ggplot2" Based Plots with Statistical Details. CRAN Available online: https://cran.r-project.org/web/packages/ggstatsplot/index.html (accessed on Nov 2, 2020).
16. Li, H.; van der Leun, A.M.; Yofe, I.; Lubling, Y.; Gelbard-Solodkin, D.; van Akkooi, A.C.J.; van den Braber, M.; Rozeman, E.A.; Haanen, J.B.A.G.; Blank, C.U.; et al. Dysfunctional CD8 T Cells Form a Proliferative, Dynamically Regulated Compartment within Human Melanoma. *Cell* **2018**, 1–15, doi:10.1016/j.cell.2018.11.043.
17. Boland, C.R.; Goel, A. Microsatellite Instability in Colorectal Cancer. *Gastroenterology* **2010**, *138*, 2073, doi:10.1053/j.gastro.2009.12.064.
18. Young, M.D.; Mitchell, T.J.; Vieira Braga, F.A.; Tran, M.G.B.; Stewart, B.J.; Ferdinand, J.R.; Collord, G.; Botting, R.A.; Popescu, D.M.; Loudon, K.W.; et al. Single-cell transcriptomes from human kidneys reveal the cellular identity of renal tumors. *Science (80-.).* **2018**, *361*, 594–599, doi:10.1126/science.aat1699.
19. Grasso, C.S.; Tsoi, J.; Onyshchenko, M.; Abril-Rodriguez, G.; Ross-Macdonald, P.; Wind-Rotolo, M.; Champhekar, A.; Medina, E.; Torrejon, D.Y.; Shin, D.S.; et al. Conserved Interferon-γ Signaling Drives Clinical Response to Immune Checkpoint Blockade Therapy in Melanoma. *Cancer Cell* **2020**, *38*, doi:10.1016/j.ccell.2020.08.005.

© 2020 by the authors. Licensee MDPI, Basel, Switzerland. This article is an open access article distributed under the terms and conditions of the Creative Commons Attribution (CC BY) license (http://creativecommons.org/licenses/by/4.0/).
